# Cardiovascular and vasomotor pulsations in the brain and periphery during awake and NREM sleep in a multimodal fMRI study

**DOI:** 10.3389/fnins.2024.1457732

**Published:** 2024-10-08

**Authors:** Johanna Tuunanen, Heta Helakari, Niko Huotari, Tommi Väyrynen, Matti Järvelä, Janne Kananen, Annastiina Kivipää, Lauri Raitamaa, Seyed-Mohsen Ebrahimi, Mika Kallio, Johanna Piispala, Vesa Kiviniemi, Vesa Korhonen

**Affiliations:** ^1^Department of Diagnostic Radiology, Oulu Functional NeuroImaging (OFNI), Oulu University Hospital, Oulu, Finland; ^2^Research Unit of Health Sciences and Technology, Faculty of Medicine, University of Oulu, Oulu, Finland; ^3^Medical Research Center (MRC), Oulu University Hospital, Oulu, Finland; ^4^Clinical Neurophysiology, Oulu University Hospital, Oulu, Finland

**Keywords:** sleep, cardiovascular pulsations, vasomotor pulsations, heart rate variability, ultrafast fMRI, multimodal imaging

## Abstract

**Introduction:**

The cerebrospinal fluid dynamics in the human brain are driven by physiological pulsations, including cardiovascular pulses and very low-frequency (< 0.1 Hz) vasomotor waves. Ultrafast functional magnetic resonance imaging (fMRI) facilitates the simultaneous measurement of these signals from venous and arterial compartments independently with both classical venous blood oxygenation level dependent (BOLD) and faster arterial spin-phase contrast.

**Methods:**

In this study, we compared the interaction of these two pulsations in awake and sleep using fMRI and peripheral fingertip photoplethysmography in both arterial and venous signals in 10 healthy subjects (5 female).

**Results:**

Sleep increased the power of brain cardiovascular pulsations, decreased peripheral pulsation, and desynchronized them. However, vasomotor waves increase power and synchronicity in both brain and peripheral signals during sleep. Peculiarly, lag between brain and peripheral vasomotor signals reversed in sleep within the default mode network. Finally, sleep synchronized cerebral arterial vasomotor waves with venous BOLD waves within distinct parasagittal brain tissue.

**Discussion:**

These changes in power and pulsation synchrony may reflect systemic sleep-related changes in vascular control between the periphery and brain vasculature, while the increased synchrony of arterial and venous compartments may reflect increased convection of regional neurofluids in parasagittal areas in sleep.

## Introduction

Angelo Mosso demonstrated in 1880 that both cerebral blood flow level and cardiovascular brain pulsations increased after brain activation in a patient with a cranial defect (Mosso, 1880). The same increases in flow and pulsatility can now be non-invasively detected using ultrafast 10 Hz whole-brain functional magnetic resonance imaging (fMRI) measurements, thus allowing sufficient temporal resolution of each arterial impulse, without any aliasing (Kiviniemi et al., 2016; Huotari et al., 2019).

The ultrafast whole-brain scanning with magnetic resonance encephalography (MREG) also enables the simultaneous detection of two distinct contrast sources of brain fluid dynamics (Kiviniemi et al., 2016); in addition to the classical blood oxygenation level dependent (BOLD), each arterial impulse presents a negative signal dip due to momentary spin phase decoherence (von Schulthess and Higgins, 1985; Ogawa and Lee, 1990; Duyn, 1997; Buxton, 2012). This allows the dissociation of cerebral arterial impulses from slow venous BOLD signals due to (i) their differing, i.e., spin phase vs. susceptibility-based magnetic resonance (MR) signal generation mechanisms, and (ii) their differing time and frequency domains (Kiviniemi et al., 2016; Huotari et al., 2019; Raitamaa et al., 2021). Setting a cardiovascular hemodynamic envelope (CHe) over each arterial impulse peak allows the detection of the local vasomotor arterial dilations, which closely follow brain activations as directed by neurovascular coupling (Huotari et al., 2022). The CHe signal was shown to precede the venous BOLD signal by approximately 1.3 s in visual cortical areas (Huotari et al., 2022). Also, breath-hold stimulation caused changes in the respiratory brain stem CHe signal (Raitamaa et al., 2019).

During sleep, the amplitude of the cardiovascular impulses and of the three known physiological brain pulsations, namely very low-frequency (VLF < 0.1 Hz), respiration, and cardiac, increase in power along with the transition of brain electrophysiological signals towards slow delta electroencephalography (EEG) oscillations (Helakari et al., 2022). The increased amplitude in these various pulsations drives blood and cerebrospinal fluid (CSF) flow to maintain brain homeostasis. In particular, cardiovascular pulsations have been shown to drive the transfer of solutes along periarterial CSF spaces via interstitial fluid (ISF) spaces into perivenous conduits (Iliff et al., 2012; Nedergaard, 2013; Mestre et al., 2018; Meng et al., 2019), in what has come to be known as the glymphatic clearance pathway. At least in sub-arachnoid periarterial spaces, the main driver of hydrodynamic exchange is cardiovascular pulsations (Iliff et al., 2012; Santisakultarm et al., 2012; Mestre et al., 2018). Moreover, the slow (< 0.1 Hz) vasomotor waves of smooth muscle tone in arterial walls of mice impact the brain solute convection along the blood vessel basement membranes (van Veluw et al., 2020). The basement membrane, which extends into the interstitial space, can serve as the exchange route for solute trafficking both in periarterial and perivenous spaces (Aldea et al., 2019).

The investigation of the relation between the pulsations in both arterial and venous compartments allows a new perspective on understanding the physiology of cerebral blood flow and solute transport. Synchrony between the pulses of these two compartments could reflect the state of the conduits linking the arterial and venous sides; increased synchrony could be related to increased flow conductance between the compartments, whereby arterial flow changes would increasingly drive venous clearance. Furthermore, larger arterial impulse power supports the idea of increased convection of solutes, which is characterized by increased forward speed and reduced backward flow pulsations in pial periarterial spaces (Mestre et al., 2018).

In this study, we used an ultrafast 10 Hz fMRI MREG sequence to investigate sleep-induced changes in the synchrony between peripheral and brain pulsations, and between intracranial blood compartment pulsations in healthy volunteers. We thereby tested the hypothesis that the increased power of the pulsations during sleep would synchronize the interaction between the venous and arterial spaces as compared to the awake state. At first, we (1) confirmed the accuracy of MREG for detecting pulsations against the peripheral fingertip photoplethysmography (PPG). We then investigated the correlation between (2) intracranial cerebral arterial vasomotor wave (CHe) and the venous-derived brain BOLD signal, (3) peripheral blood volume changes and the brain venous BOLD signal, and (4) peripheral arterial vasomotor activity and the brain venous BOLD signal during awake and EEG-verified sleep.

## Materials and methods

### Subjects

The analysis was performed on 10 healthy volunteers (age in years: 25.5 ± 3.4, 5 females). The study was approved by the Regional Ethics Committee of the Northern Ostrobothnia Hospital District and was performed in Oulu, Finland. Written informed consent was obtained from all participants according to the Declaration of Helsinki. All subjects were healthy and met the following inclusion criteria: no continuous medication, no neurological nor cardio-respiratory diseases, non-smokers, and no pregnancy. The subjects were instructed not to consume caffeine during the 4 h before the awake scan session and 8 h before the sleep scan session. Participants agreed to abstain from alcohol consumption 12 h before the scans.

### Data acquisition

The subjects participated in two separate measurement sessions 3 days apart, with an awake scan session in the afternoon and a sleep scan session in the early morning after 24 h of sleep deprivation. Participants wore an Ōura Ring^1^ for at least 24 h preceding both imaging sessions in a Siemens MAGNETOM 3 T Skyra scanner (Siemens Healthineers AG, Erlangen, Germany) equipped with a 32-channel head-coil. We used the ultrafast fMRI imaging sequence MREG, consisting of a 3D single shot stack of spiral sequences that under-samples k-space, which allows sampling of physiological pulsations at 10 Hz (Assländer et al., 2013). The following scanning parameters were used for MREG: repetition time (TR = 100 ms), echo time (TE = 36 ms), field of view (FOV = 192 mm), flip angle (FA = 5°), and 3 mm isotropic cubic voxel. MREG scan images were reconstructed using L2-Tikhonov regularization with lambda 0.1, with the latter regularization parameter having been determined by the L-curve method using a MATLAB recon-tool provided by the sequence developers (Hugger et al., 2011). Anatomical 3D structural T1 MPRAGE (TR = 1900 ms, TE = 2.49 ms, FOV = 240 mm, FA = 9° and 0.9 mm slice thickness) images were used to register MREG datasets into Montreal Neurological Institute (MNI) space.

**Figure 1:**
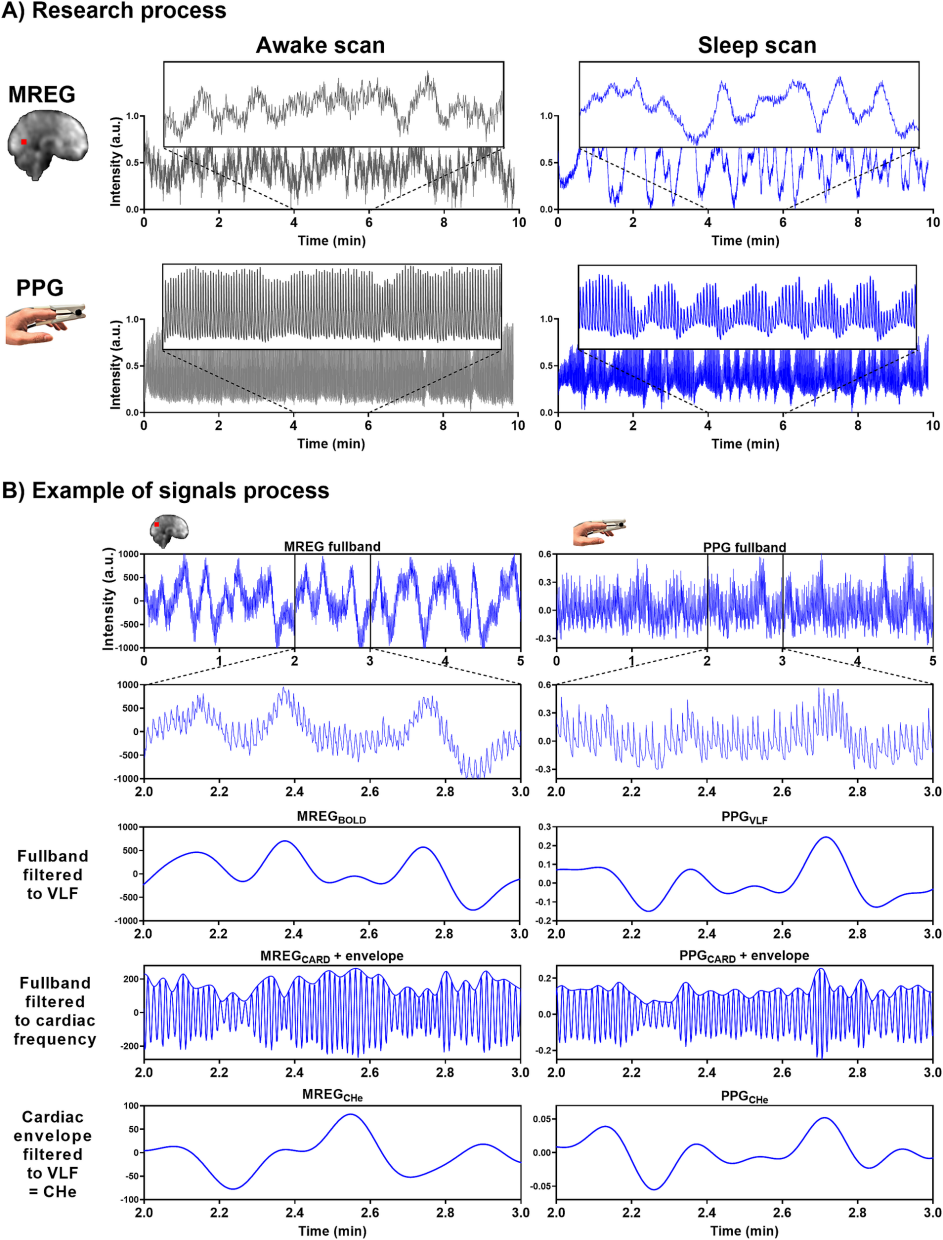
Representative signals for awake and NREM sleep. **(A)** During the awake scan (10 min) eyes were open and during the sleep scans (2×10 min) subjects were allowed to fall asleep. MREG and PPG signals are from an awake (5% sleep, EEG scored) subject and from a sleeping (85% sleep, EEG scored) subject. Example signals are normalized. **(B)** In the analysis we used 5-min epoch signals. Examples of MREG and PPG signal processing in a sleeping (30% NREM N1 and 70% NREM N2 EEG scored sleep) subject.

During the awake scan session, one 10-min resting state MREG sequence was taken with eyes open and gaze fixated on a cross projected on a monitor. During the sleep measurement session 3 days later, two 10-min MREG scans were first taken. Before the actual fMRI scan, sleep-deprived participants were instructed to lie down on the scanner bed, close their eyes, and attempt to sleep, accompanied by the dimming of lights in the MRI room. An anatomical T1-weighted scan was performed at the end of both sessions. Part of the research pipeline is shown in Figure 1, with greater details presented in Helakari et al. (2022).

Fingertip peripheral PPG, and end-tidal-CO_2_ (EtCO_2_) were measured in synchrony using a 3 T MRI-compatible anesthesia monitor (Datex-Ohmeda S/5 Collect software), as described previously (Korhonen et al., 2014). Cuff-based blood pressure of each subject was also measured sitting and supine before both scanning sessions. The PPG probe was placed on the left index finger, with data acquisition at 300 Hz. Right index fingertip PPG data were collected using the MR scanner for verification purposes.

### Pre-processing

Preprocess steps and analyses were performed using MATLAB (R2020b, The MathWorks, Natick, MA), Functional MRI of the Brain Software Library (FSL; Brain Extraction Tool (BET), version 5.09) (Smith, 2002; Jenkinson et al., 2012) and Analysis of Functional NeuroImages (AFNI; version 2) (Cox, 1996). As a part of quality control, we visually inspected the PPG data. We identified and corrected high peaks caused by motion artifacts by despike, allowing the use of the mean value for adjacent PPG amplitudes during analysis.

FSL BET was used for brain extraction with neck and bias-field correction from structural 3D MPRAGE volumes (Smith, 2002). MREG datasets passed through a typical FSL preprocessing pipeline (Jenkinson et al., 2012), with high-pass filtering at a cutoff of 0.008 Hz. Datasets were spatially smoothed with 5 mm Full Width at Half Maximum Gaussian kernel. AFNI *3dDespike* was used to remove spikes from the datasets caused by head movements. Head motion correction was carried out using FSL 5.08 MCFLIRT (Functional MRI of the Brain Linear Image Registration Tool) (Jenkinson et al., 2002). MCFLIRT relative or absolute mean displacement values (awake_rel_ = 0.021 ± 0.004 mm, sleep_rel_ = 0.021 ± 0.004 mm, awake_abs_ = 0.27 ± 0.18 mm, sleep_abs_ = 0.16 ± 0.07 mm) or calculated each subject’s mean frame-wise displacement (awake_fd_ = 0.019 ± 0.004 mm, sleep_fd_ = 0.021 ± 0.005 mm) did not differ significantly between awake and sleep states (p_rel_ = 0.46, p_abs_ = 0.07, p_fd_ = 0.35).

EEG was recorded using the Electrical Geodesics MR-compatible GES 400 system (Magstim), with a 256-channel high-density scalp net. Electrode impedances were < 50 kΩ and the sampling rate was 1 kHz. Signal quality was tested outside the scanner room by recording 30-s epochs of EEG with eyes open and eyes closed. EEG recordings were preprocessed using the Brain Vision Analyzer (Version 2.1; Brain Products) after converting to a compatible format via BESA Research (Version 7.0). Gradient artifacts due to static and dynamic magnetic fields during the MRI data acquisition, along with the ballistocardiograph (BCG) artifacts, were corrected using the average artifact subtraction method (Allen et al., 1998, 2000). The data was checked to ensure the absence of gradient and BCG artifacts.

### Cardiorespiratory analysis

**Figure 2:**
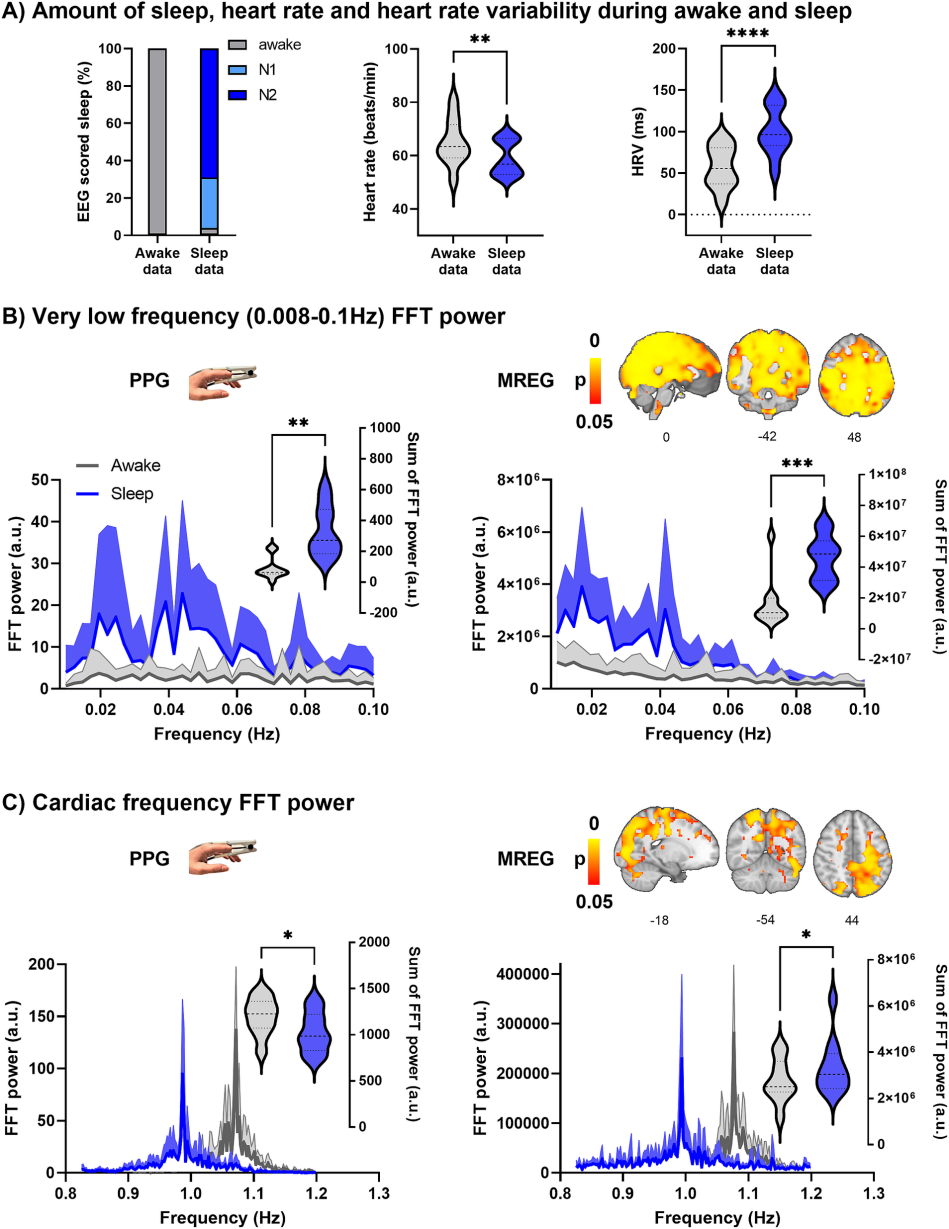
Prevalence of vigilance state in the sleep data and spectral FFT power of fingertip peripheral PPG and MREG recordings during awake and sleep states (*n* = 10). **(A)** During the sleep recordings, subjects slept (NREM N1-N2 sleep) 96% of the time. Calculated from PPG, the heart rate (HR, *p* = 0.0099 **) was significantly lower during sleep, and heart rate variability (HRV, *p* < 0.0001 ****) significantly higher during sleep compared to awake. **(B)** Group average (+standard deviation) PPG and global MREG spectra in very low-frequency (VLF, 0.008–0.1 Hz) show that the VLF power was significantly higher during sleep compared to awake in PPG (*p* = 0.0014, **) and in global MREG data (*p* = 0.0007, ***). **(C)** The frequency-normalized group mean cardiac frequency spectra showed that cardiac frequency power was significantly lower in fingertip PPG (*p* = 0.037, *) but higher in global MREG (*p* = 0.035, *) during sleep compared to awake.

To get the maximum amount of awake signal from awake scans and likewise, NREM N2 sleep from sleep scans, PPG, EtCO_2,_ and MREG signals were sectioned into continuous simultaneous 5-min segments using MATLAB and *fslroi* (Figures 1B, 2A).

PPG and EtCO_2_ signals from an anesthesia monitor were used to verify group cardiorespiratory parameters based on the individual ranges of each subject. Heart rate (HR) from the PPG signal and respiratory rate (RR) from the EtCO_2_ signal were determined using the MATLAB *findpeaks* function with subject-specific input parameters. RR of one subject was determined from MREG data (Järvelä et al., 2022; Kananen et al., 2022) due to invalid respiration data. Heart rate variability (HRV) was calculated from the PPG signal to determine the root mean square of successive differences between normal heartbeats (Shaffer et al., 2014) for awake and sleep scans. HR, HRV, and RR were calculated from one 5-min awake scan and one 5-min sleep scan, which were selected for the analysis.

### Power analysis of PPG and global MREG

The VLF range of 0.008–0.1 Hz was chosen to get the maximum possible coverage of the low frequencies without crosstalk with respiratory frequencies. 5-min PPG data were down-sampled to 10 Hz using MATLAB to analyze fast Fourier transform (FFT) power and cross-correlate with MREG data.

Cardiac frequency ranges were obtained from individual PPG spectrums without respiratory aliasing, harmonics, or noise for correlation analysis. After analysis, the data were registered into MNI space at 3 mm resolution to enable comparable analysis between awake and sleep datasets.

The down-sampled 5-min PPG signals were normalized using Z-score normalization (Gasparini et al., 2022) and MATLAB *fft* function was used to calculate the respective FFT power spectra. Z-score normalization ensured that the amplitudes of the PPGs were consistent. Global FFT power density maps were calculated using AFNI *3dPeriodogram* function for 5 min of full band (0.008–5 Hz) MREG data, followed by application of the *fslmeants* function to calculate the average FFT spectrum of all brain voxels. The VLF and individual cardiac FFT power were calculated using AFNI *3dTstat*. The VLF and cardiac band powers and mean (+ standard deviation) power spectra were visualized using GraphPad Prism 9 software. The most prominent individual cardiac peaks were shifted to the group mean frequency of both awake and sleep scans, and the mean power spectrum was calculated for each condition.

### Cross-correlation analysis

The voxel-wise positive maximum cross-correlation coefficients (CC_MAX_) between the pre-processed PPG and MREG signals in awake and sleep states were calculated using the MATLAB *xcorr* function with sequence normalization *‘coeff’* and the corresponding time delays yielding the maximum correlation. We restricted the search range of time delays to be within ±10 s, as the total transit time of blood through the head is less than 10 s in healthy subjects (Schreiber et al., 2002). Cross-correlation was performed between the following 5-min signals:

1. **Validation of MREG cardiac signal using PPG cardiac signal: Fingertip cardiac pulsatility to cerebral cardiac pulsatility correlation.** Cardiac signals reflected the resultant arterial pulsation. Here, the PPG signal was first analyzed with FFT and was then band-pass filtered to each subject’s individual cardiac frequency band (Järvelä et al., 2022) using MATLAB. First, the raw MREG data were multiplied by −1 to raise the R-peak. Next, using the individual frequency information, the full-band MREG data were band-pass filtered to each subject’s cardiac range using the AFNI *3dTproject* to obtain signal without noise, respiratory aliasing, or harmonic cardiac waves. These cardiac-filtered signals are henceforth designated as PPG_CARD_ and MREG_CARD_.
2. **Investigating the correlation of intracranial cerebral arterial vasomotor wave (CHe) to the venous-derived brain BOLD signal.** The brain MREG signal contains two forms of very low-frequency (VLF, 0.008–0.1 Hz) vascular waves in addition to other physiological signals; there is an active *arterial* vasomotor tone wave, which is followed some 1.3 s later by a passive *venous* wave resulting from downstream ballooning due to blood volume and oxygenation changes (Huotari et al., 2022). Since these two waves have a different MR contrast origin (arterial spin phase vs. venous susceptibility), they can be separated from ultrafast MREG data with the following procedure: Cerebral MREG_CHe_ reflecting *arterial vasomotor tone waves* can be obtained from peak-to-peak cardiovascular impulse amplitudes using a cardiovascular hemodynamic envelope (MREG_CHe,_ c.f. Huotari et al., 2022). The MREG_CHe_ were obtained by setting an envelope over the individually verified cardiac frequency MREG_CARD_ signal peaks with MATLAB *findpeaks*, *spline*, and *ppval* functions. The classical BOLD baseline signal reflecting venous blood volume and (de)oxygenation level fluctuations were obtained, importantly also without aliased physiological noise, harmonics, and modulations, by bandpass filtering the full band MREG data to the VLF range using AFNI *3dTproject*; we henceforth designate this as MREG_BOLD_. To analyze similar physiological phenomena, the MREG_CHe_ data were also bandpassed to the *identical (0.008–0.1 Hz)* VLF range. Finally, the MREG_CHe_ vs. MREG_BOLD_ were cross-correlated on a voxel-by-voxel basis.
3. **Investigating the correlation of peripheral blood volume changes to the venous BOLD signal.** Fingertip PPG signals reflect blood volume changes (Allen, 2007; Abay and Kyriacou, 2018). The PPG signal was bandpass filtered to the VLF band using MATLAB, which we designate as the peripheral PPG_VLF_; this was also cross-correlated to MREG_BOLD_.
4. **Investigating the correlation of peripheral arterial vasomotor activity to the brain venous BOLD signal.** Peripheral vasomotor tone (a.k.a. arterial amplitude) waves were also estimated from the fingertip PPG signal using the CHe technique as described above in item 2. The PPG signal was bandpass-filtered to the individual cardiac range using MATLAB to obtain a signal uncontaminated with other physiological signals. An envelope was set above the cardiac peaks for extraction of the PPG_CHe_ similar as above. Finally, the envelopes were bandpass filtered to the VLF range for correlation analysis.

### Analysis of EEG data

The EEG data were scored in 30 s epochs by two experienced specialists in clinical neurophysiology following the American Academy of Sleep Medicine (AASM) 2017 guidelines, and the final sleep state scoring was obtained by consensus of both specialists. Using established criteria, EEG epochs were scored as wake, NREM N1 (light sleep), NREM N2 (intermediate sleep with sleep spindles and/or K-complexes), NREM N3 (slow wave sleep), or REM (sleep with rapid eye movements).

### Ōura ring activity data

The subject sleep/wake status was monitored with the multisensory Ōura Ring sleep tracker (see foonote 1) on the days before each scan, with a detailed analysis of the final 24 h before scanning. The principles and more results of the ring data are presented in Helakari et al. (2022).

### Statistical analysis

Whole brain voxel-wise comparisons between different awake/sleep states in the same subjects were performed by two-sample t-test using a paired nonparametric threshold-free permutation test (5,000 permutations) implemented in *vlisa_2ndlevel* from LIPSIA (Lohmann et al., 2018). The Shapiro–Wilk test was used to examine the normality of the distribution of the variables. For the sum of PPG FFT power, Ōura ring activity data, cardiorespiratory signals, and head motion, we calculated statistical differences by two-sided paired Student’s t-test using GraphPad Prism 9. Results are reported as mean ± standard deviation, and *p*-values below 0.05 are considered significant (*p* < 0.05: *, *p* < 0.01: **, *p* < 0.001: ***, *p* < 0.0001: ****).

## Results

We investigated cerebral and peripheral cardiovascular pulsatility, as well as vasomotor waves, and examined their relationships during awake and NREM sleep epochs. Subjects were scanned after a monitored normal night sleep and after monitored sleep deprivation (24 h) time. Based on Ōura recordings, subjects slept 7.8 ± 1.7 h (N3 sleep 1.0 ± 0.5 h) during normal night and slept 15 ± 25 min (no N3 sleep) during deprivation time (*p* < 0.0001 ****). Ōura-measured deprivation time was 24.2 ± 1.4 h.

During the awake scan, subjects were awake for 100% of the five-minute data (Figure 2A), whereas during the sleep scan, subjects were awake for 4 ± 10% of the five-minute data (Figure 2A), with 27 ± 31% N1 sleep and 69 ± 31% N2 sleep, but no N3 or REM sleep was detectable.

### Cardiorespiratory analysis

Cardiorespiratory parameters were calculated from the fingertip PPG and EtCO_2_ signals from awake and sleep five-minute epochs (Table 1).

**Table 1:** Cardiorespiratory parameters.

Outcome	N	Awake data	Sleep data	Mean change (95% CI)	*P*-value
		Mean (SD)	Mean (SD)		
Heart rate (b/min)	10	65 (9)	59 (7)	−6 (−10 to −2)	0.0099^#^
Heart rate variability (ms)	10	57 (25)	100 (29)	43 (29 to 57)	<0.0001^#^
Respiratory rate (b/min)	10	16.3 (3.3)	15.1 (2.4)	−0.9 (−2.5 to 0.6)	0.21^#^
Cardiac frequency width (Hz)	10	0.24 (0.05)	0.36 (0.04)	0.12 (0.08 to 0.17)	0.0001^#^

^#^Paired t-test. SD = Standard deviation. CI = Confidence interval.

Heart rate (HR) decreased (*p* = 0.0099, **, Figure 2A) and heart rate variability (HRV) increased (*p* < 0.0001, ****, Figure 2A) significantly during sleep compared to awake. On average, males have lower HR (Male/Female awake 62/68 b/min, sleep 55/63 b/min) and HRV (M/F awake 51/63 ms, sleep 90/111 ms) than females but not statistically significant. The width of the cardiac spectrum expanded during sleep (*p* = 0.0001, ***). Respiratory rate (RR) decreased in NREM sleep (M/F awake 16.3/16.2 b/min, sleep 14.4/15.9 b/min) but showed no significant differences between sleep and awake recordings. Group mean blood pressure (systole/diastole) was 127/77 mmHg (sitting), 127/69 mmHg (supine) before the awake scan, and 127/75 mmHg (sitting), 125/68 mmHg (supine) before the sleep scan.

### Power analysis of PPG and global MREG

FFT amplitude power was calculated from the fingertip peripheral PPG signal and global MREG signal from awake and sleep data (Figures 2B,C). The FFT spectra of both the fingertip and the global brain signals show the greatest increase in power during sleep at 0.02 Hz and 0.04 Hz VLF frequencies. FFT power was significantly higher in the VLF band during sleep compared to awake in PPG (*p* = 0.0014, **) and global MREG (*p* = 0.0007, ***), much as reported previously (Helakari et al., 2022). In cardiac frequency, the FFT power was significantly lower in fingertip PPG (*p* = 0.037 *), but interestingly was higher in the brain global MREG (*p* = 0.035, *) during sleep compared to awake.

### Beat-to-beat cardiovascular synchrony between periphery and brain

**Figure 3:**
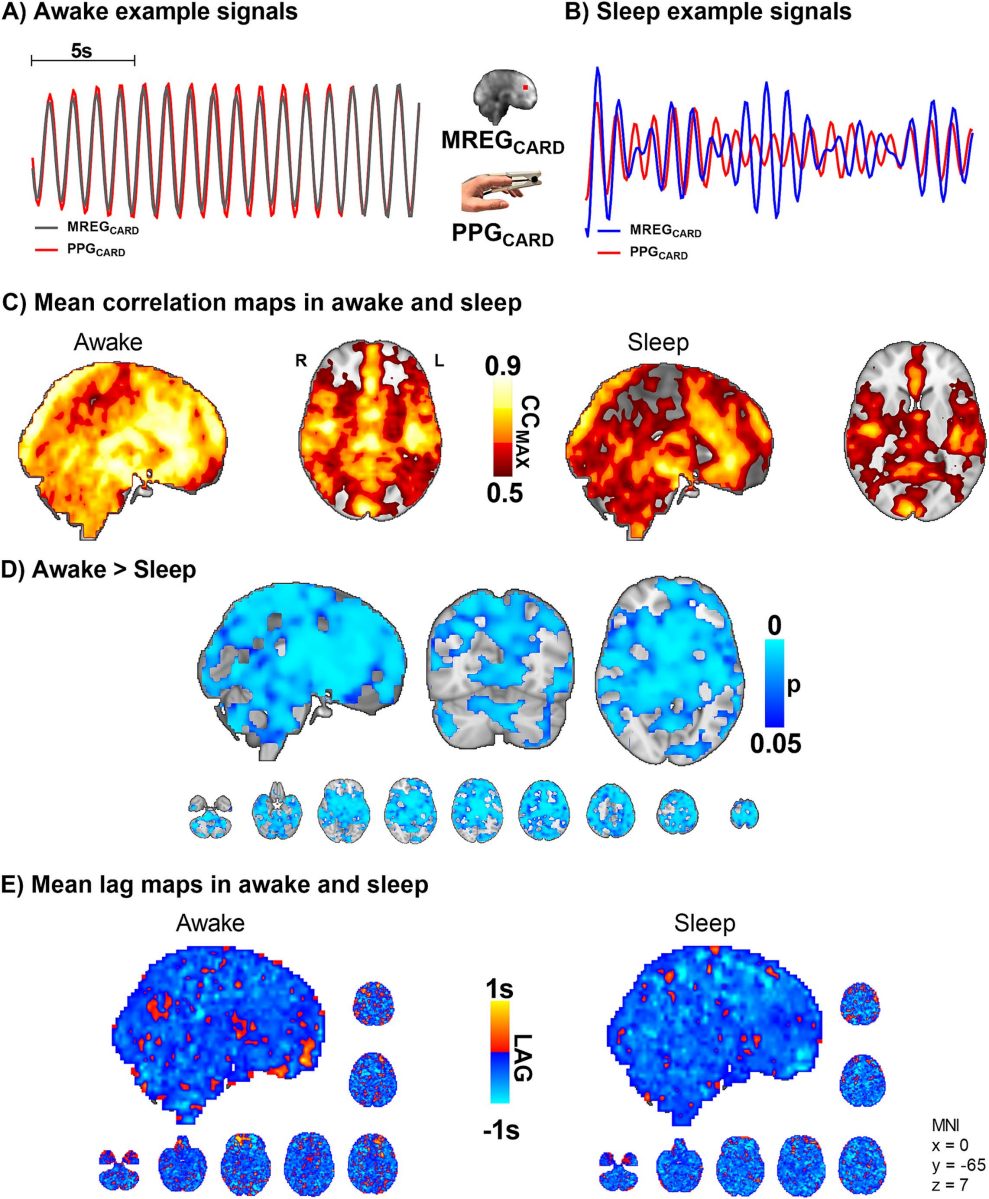
Sleep decreases the correlation between peripheral and brain cardiovascular measures of beat-to-beat pulses (*n* = 10). **(A,B)** Representative fingertip and brain MREG cardiac signals during awake and sleep states. **(C,D)** The group level maximum cross-correlation coefficients (CC_MAX_) between peripheral PPG_CARD_ and brain MREG_CARD_ during awake were nearly identical (CC_MAX_ ~ 0.9) over most of the brain. Sleep reduced the beat-to-beat correlations significantly compared to awake state (*p* < 0.05), extending virtually over the whole brain. **(E)** Lag between the signals indicated brain cardiovascular impulse preceding the peripheral as impulse propagates first into the brain. The lag did not change significantly during sleep.

PPG and MREG signals were filtered to the individual cardiac range. Figure 3 illustrates the drastic amplitude modulation and de-synchronization in sleep between the measurement points. As a sign of high temporal accuracy, the MREG_CARD_ signal of the brain gave nearly identical pulsation signals compared to PPG_CARD_, especially in the proximity of major arteries, c.f. Figure 3C. However, during sleep, the CC_MAX_ between peripheral PPG_CARD_ and brain MREG_CARD_ were significantly lower compared to the awake scan, extending over most parts of the brain and especially in central areas (*p* < 0.05, Figure 3D). Group mean lag values ranged between −1 - +1 s during awake recordings, and without any significant change in sleep (Figure 3E). The MREG_CARD_ lead the fingertip lags by approximately 0.3 ± 0.4 s in awake scans and 0.4 ± 0.3 s in sleep scans.

Based on the above results, the cerebral vasoconstrictions may modulate cardiovascular pulsation, as reflected in the amplitude, more in sleep than while awake. Vasomotor contractions narrow blood vessels and increase local blood pressure (Myllylä et al., 2011), which causes more rapid impulse propagation. The speed-up dephases the temporal synchrony between peripheral and brain cardiovascular signals (Figures 3A–D). As the vasomotor tone relaxation does the opposite (slows down), the overall change in the averaged lag structure between the signals over the whole brain remains unchanged (Figure 3E).

**Figure 4:**
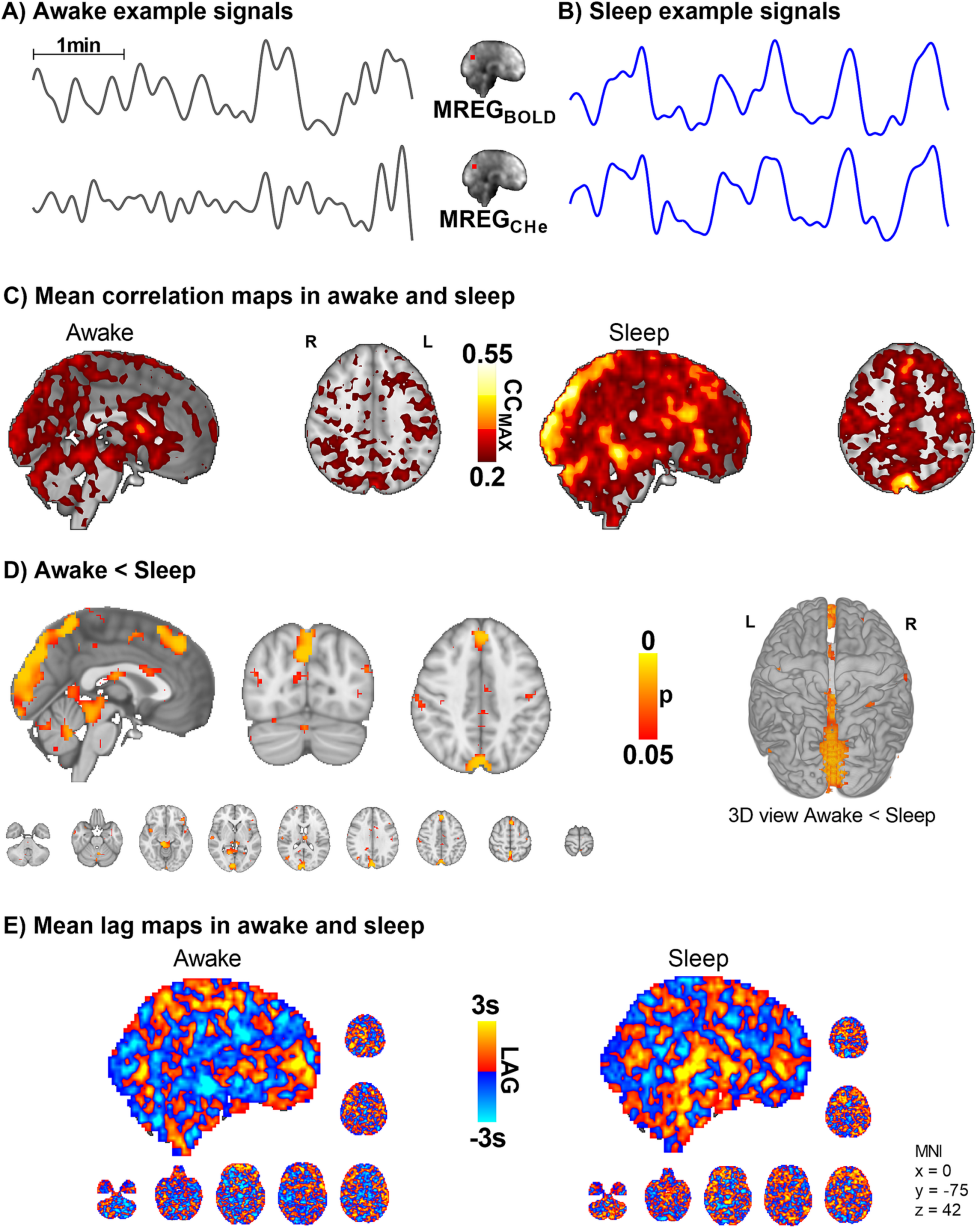
Intracranial synchrony of VLF modulations of arterial MREG_CHe_ pulsations and venous MREG_BOLD_ signals increases in sleep (*n* = 10). **(A,B)** Example signals of MREG_CHe_, and MREG_BOLD_ during awake and sleep recordings. **(C,D)** Cerebral signals between arterial pulsatility (MREG_CHe_) and vein-dominated baseline BOLD signal (MREG_BOLD_) harmonize during sleep, especially near large veins and the parasagittal area. **(E)** There were no changes in the complex lag structure between the VLF changes in MREG_CHe_ vs. MREG_BOLD_, which was more heterogeneous than the cardiac pulsations presented in Figure 3.

As there were strong vasomotor contractions in sleep both in peripheral and brain MREG_CARD_ signals, we proceeded to assess how the arterial vasomotor waves in the amplitude envelope correlated with the venous BOLD signal. We compared the regional brain vasomotor tone modulations with the downstream venous BOLD signal between awake and sleep states (Figure 4). In sleep, the MREG_CHe_ and MREG_BOLD_ correlations were significantly higher in the visual cortex, cerebellum, and the parasagittal area, and in proximity to large veins (whole-brain analysis, *p* < 0.05, Figure 4D). In other words, the cerebral arterial vasomotor waves and venous BOLD fluctuations become more synchronous during sleep in these regions, which were previously shown to present slow wave EEG activity reflecting increased fluid transport during sleep (Helakari et al., 2022). As before, the lag structure between these signals remained complex and unchanged by sleep (Figure 4E). Over the whole brain the average on the BOLD signal preceded the CHe signal by 0.07 ± 1.4 s in waking, whereas in sleep the CHe precedes BOLD by 0.13 ± 1.4 s.

### Peripheral blood volume vs. brain MREG_BOLD_ fluctuations

**Figure 5:**
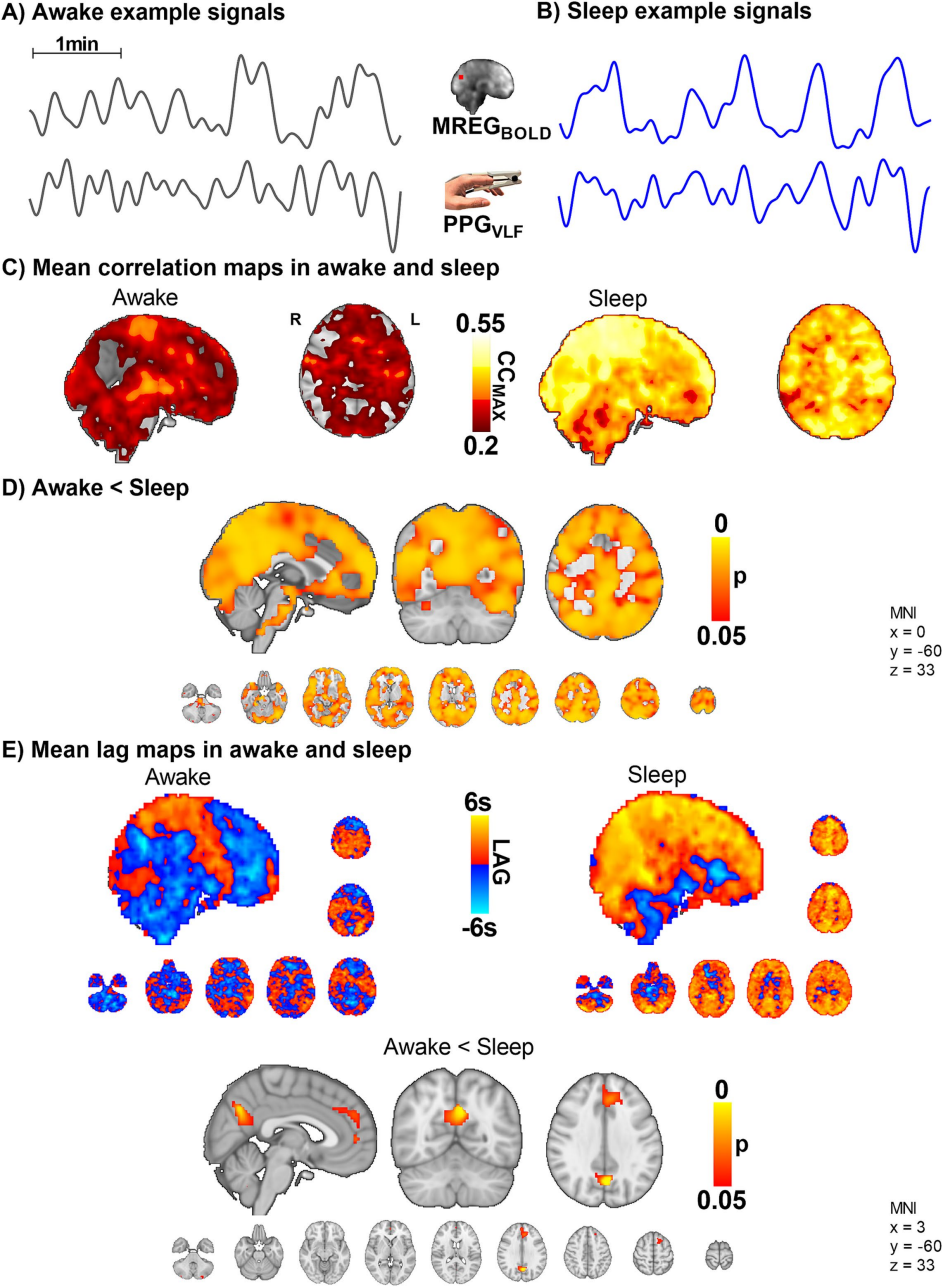
The VLF blood volume oscillations in the periphery (PPG_VLF_) and venous brain MREG_BOLD_ signal synchronize in sleep with a significant causal lag change in default mode brain areas (*n* = 10). **(A,B)** Example signals of MREG_BOLD_ and PPG_VLF_ during awake and sleep states. **(C,D)** The group level CC_MAX_ between MREG_BOLD_ and PPG_VLF_ during awake and sleep shows that correlations were significantly higher in sleep compared to awake (*p* < 0.05) widely in the brain. **(E)** In sleep, the fingertip PPG_VLF_ signal has a significantly earlier onset than the MREG_BOLD_ signal (*p* < 0.05) in the posterior cingulate gyrus, the anterior default mode network area, and cerebellum.

We next set out to examine the correlation between brain and peripheral blood volume pulsations by correlating the slow fingertip blood volume oscillations (PPG_VLF_) in periphery vs. brain MREG_BOLD_ VLF oscillations. The CC_MAX_ between PPG_VLF_ and MREG_BOLD_ was significantly higher in sleep compared to awake scans, extending widely over brain cortical regions (*p* < 0.05, Figure 5). Interestingly, in the awake state, the fingertip blood volume oscillations follow the brain signal by a mean (± SD) of 0.03 ± 1.8 s, but in sleep, the peripheral oscillation precedes the brain MREG_BOLD_ signal by 2.1 ± 1.9 s. The phase difference was significant in the posterior cingulate gyrus, anterior default mode network area, and cerebellum in sleep (Figure 5E).

### Peripheral arterial vasomotor CHe modulations vs. venous MREG_BOLD_ fluctuations

**Figure 6:**
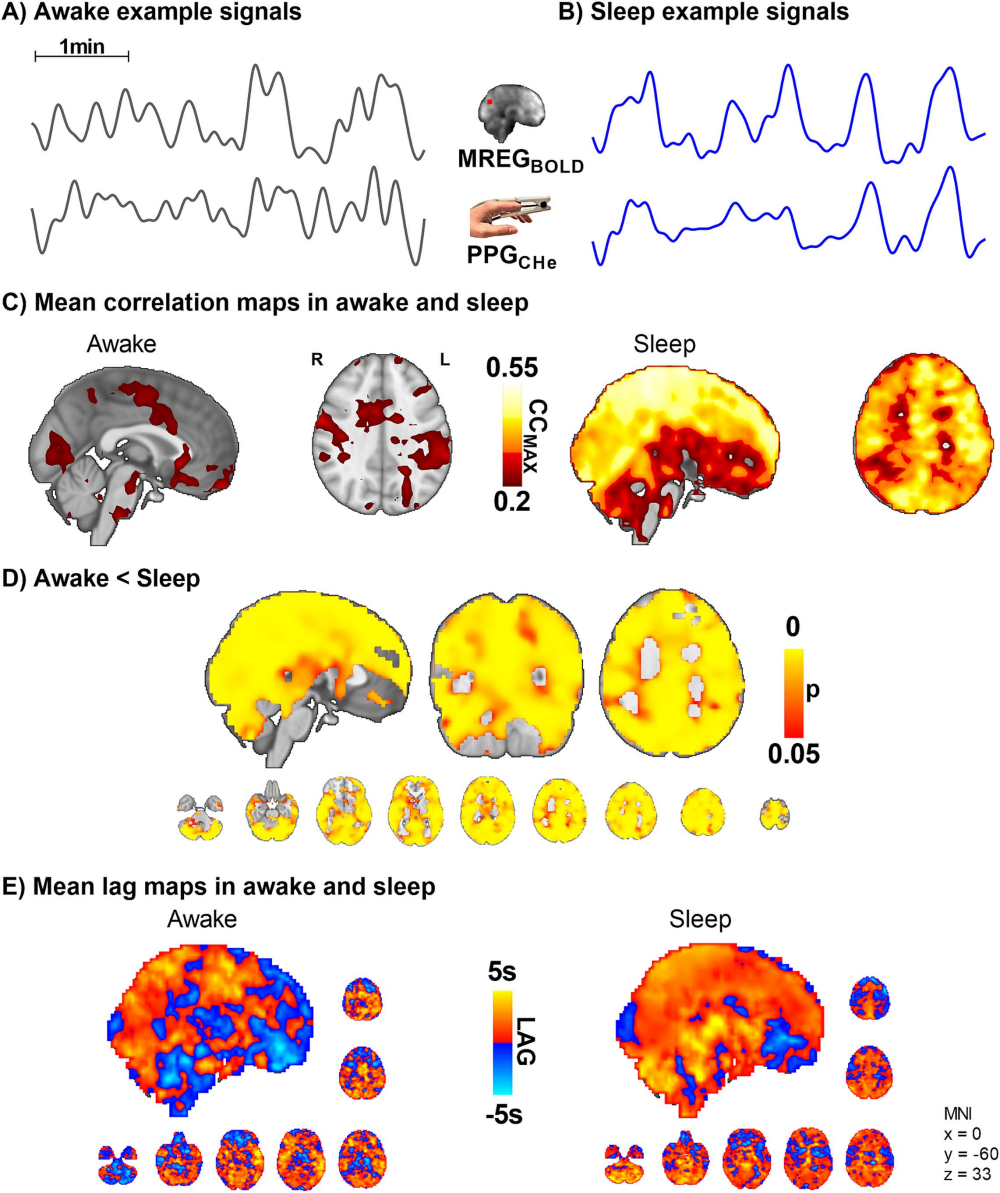
Peripheral vasomotor tone PPG_CHe_ and brain venous MREG_BOLD_ synchronize in sleep (*n* = 10). **(A,B)** Representative MREG_BOLD_ and PPG_CHe_ signals during awake and sleep states. **(C,D)** The correlation of the peripheral cardiovascular hemodynamic envelope (CHe) to the brain VLF MREG_BOLD_ signal shows that peripheral arterial vasomotor waves synchronize significantly with the cerebral BOLD signal during sleep. **(E)** The lag shows a non-significant tendency towards increased lead in the peripheral and brain waves during sleep.

To ascertain how the peripheral arterial vasomotor tone PPG_CHe_ connects with venous brain MREG_BOLD_ fluctuations in sleep we compared these two signals. While awake, the peripheral vasomotor tone did not correlate with the brain venous BOLD signal, but during sleep the correlation between fingertip PPG_CHe_ and MREG_BOLD_ was significantly higher (*p* < 0.05, Figure 6), extending over practically the whole brain. The change in the correlation between awake and sleep states was more significant than the correlation between MREG_BOLD_ vs. PPG_VLF_ signals (Figure 5D). On average, the peripheral PPG_CHe_ signal preceded brain MREG_BOLD_ signals by 0.7 ± 1.8 s in the awake state and 0.8 ± 1.6 s in sleep (Figure 6E, non-significant).

### Arterial PPG_CHe_ vs. brain arterial MREG_CHe_

We further examined the correlations between peripheral PPG_CHe_ and brain MREG_CHe_. The correlations between the CHe signals, which reflect amplitude modulations of the vasomotor tone, were highest in the arterial and sinus sagittal areas (Supplementary Figure S1). There was no significant effect of sleep on synchrony. The brain signals preceded on average the PPG_CHe_ signal by 0.6 ± 1.4 s in awake state and by 0.03 ± 1.5 s in sleep, with a non-significant tendency for reversed lead from the peripheral signal. Examining the power of PPG_CHe_ and MREG_CHe_ did not reveal any statistically significant differences between awake and sleep.

## Discussion

In this study, we assessed NREM sleep-induced changes in the relationship between arterial cardiovascular pulsatility, slow vasomotor waves, and venous blood fluctuations both inside the brain and with respect to the periphery. In sleep, the power of the VLF waves and the synchrony between the VLF waves in the periphery and brain significantly increased, while the near-perfect cardiovascular beat-to-beat impulse synchrony deteriorated. Within the brain, the arterial MREG_CHe_ slow vasomotor waves, and venous MREG_BOLD_ waves became more synchronized in parasagittal brain regions. Moreover, VLF (MREG_BOLD_) and cardiovascular (MREG_CARD_) powers in the brain were both significantly higher in sleep. In the periphery, however, the VLF power increased while the power of cardiovascular pulsations declined during sleep.

### Cardiovascular impulse synchrony between the periphery and brain vanishes in NREM sleep

The total power over the individual range of cardiac pulsation power increased in parietal brain areas, while the principal cardiac peak power was lower during sleep. In other words, the total cardiovascular power widens over wider frequency range in brain tissue during sleep, suggesting an increase in frequency modulation of the cardiovascular pulsation itself. The same phenomenon was also detected in peripheral PPG signals, also known as HRV, which is closely related to increased autonomic cardiovascular regulation in sleep (Mendez et al., 2010). Interestingly, however, our data show that the power of cardiovascular impulses changes in opposing directions in peripheral vs. brain signals (Figure 2). Moreover, in the present study, the MREG power increase showed a somewhat wider spatial distribution along the sagittal sinus than in our previous analysis (Helakari et al., 2022), probably due to a more restricted cardiac power analysis centered around the principal cardiac peak power, instead of the full cardiac range of the whole group.

Also, we show for the first time that the high synchrony of the brain and peripheral cardiovascular beat-to-beat pulsatility seen in the awake state disappears in NREM sleep, c.f. Figure 3. This loss of synchrony may be related to sympathetic nervous system activation and consequent vasomotor contraction that are present during NREM sleep (Kjaerby et al., 2022; Bojarskaite et al., 2023). Additionally, it has been shown in mice that brain tissue becomes softer under anesthesia, and brain stiffness is linearly correlated with water content (Ge et al., 2024), raising a question whether this could contribute to the observed changes in pulsatility synchrony in the human brain. Furthermore, the high blood pressure or vasomotor tone contractions also increase the speed of impulse propagation (Myllylä et al., 2011; Finnegan et al., 2021), which is then visible as phase desynchronization between peripheral and brain pulsations (Figures 3A,B).

During sleep, the cardiovascular signals showed marked vasomotor contractions, as indicated by cardiac impulse amplitude drops, and reflected as modulation of the amplitude envelope, i.e., MREG_CHe_, Figures 3–5. Since the coherence of the cardiovascular impulse amplitudes in brain MREG_CHe_ vs. peripheral PPG_CHe_ did not significantly differ between sleep and awake states, the desynchronization of the time domain signals must stem from signal dephasing. Thus, the vasomotor tone changes may be stronger in the brain than in the peripheral tissue. As the correlation values of the peripheral PPG (arterial tone waves and blood volume, Figures 5, 6) and brain slow waves reached a modest correlation coefficient of 0.55, and nearly perfect synchrony with a correlation of 0.9 in awake cardiovascular impulses (Figure 3), there may be currently unidentified sources of modulation. An intriguing option for such modulatory interaction is the default mode brain network which is known to reflect changes in consciousness (Kiviniemi et al., 2005). Here the default mode network BOLD signal started to follow the peripheral vasomotor CHe waves by some 2.1 s (Figure 5E) in sleep and thus, the default mode seems to be connected to the modulation of the increased vasomotor waves of the body.

### Arterial vasomotor wave—CHe

The detection of cardiovascular signals with MREG has been previously shown to be very accurate; standard physiological measurement methods have shown virtually one-to-one correlation between MREG vs. PPG in multiple datasets (Tuovinen et al., 2020; Järvelä et al., 2022). Also in this study, the correlation between PPG and MREG was again 0.9 in arterial areas of the resting state awake scan, strongly indicating that individual cardiac filtered MREG signals reflect the arterial physiology especially close to the cerebral arteries. The envelope laid over the individual cardiovascular peaks reflects the amplitude changes of successive cardiovascular pulses locally.

In terms of MR signal physics, each arterial impulse induces a fast drop in T2* weighted MREG signal due to a sudden pressure-induced disturbance in water spin precession- and phase (von Schulthess and Higgins, 1985; Duyn, 1997). In the veins, however, the dominating signal source has shown to be the *slow* susceptibility-weighted signal where the change in the venous Hb/HbO_2_ ratio increases T2* weighted signal 3–5 s after the activation-induced vasodilation (Ogawa and Lee, 1990; Buxton, 2012). As the arteries vasodilate first and the venous signal follows, these both effects can be separated using the fast scanning technique, such as the 10 Hz MREG (Raitamaa et al., 2019; Huotari et al., 2022). MREG indicated a 1.3 s time difference of the arterial dilation vs. venous BOLD signal elevation after cued visual brain activation (Huotari et al., 2022).

As the preceding arterial vasomotion (CHe) and the downstream venous BOLD signal become more similar locally in sleep, an increase in their synchrony might reflect two things: it could be a more direct flow effect from the arterial blood and/or perivascular CSF toward the venous compartments. The other explanation could be that a reduction in the recently detected spontaneous hypoxic pocket activity (Beinlich et al., 2024) in the perivenous areas during sleep, which then makes the venous BOLD signal more similar to arterial vasomotion. However, as the blood flow usually reduces in NREM sleep (Al-Shama et al., 2024), the more likely explanation is the increased flow effect drives from arterial to venous compartment, since the hypoxic events increase in slower flow. This, however, needs to be verified with *in vivo* experiments.

The increased synchrony between arterial and venous signals suggests a more harmonized exchange of fluids between these compartments. This could mean that as the arterial blood pulses into the brain, the venous blood outflow is more closely timed or matched to this inflow. Such harmonization could enhance the efficiency of fluid movement (and waste clearance) in the brain. During sleep, the brain’s fluid dynamics change in a way that makes the movement of blood and other fluids more synchronized between the arterial and venous compartments. This phenomenon, detected by the MREG technique, may have significant implications for understanding how the brain maintains its function and health during sleep.

We wanted to investigate how the hemodynamic coupling between the intracranial arterial MREG_CHe_ and venous MREG_BOLD_ signals differs between awake and sleep states. The synchrony between the arterial MREG_CHe_ and venous MREG_BOLD_ signal seen in the awake recordings was significantly higher in parasagittal areas during NREM sleep (Figure 4). Around these parietal parasagittal areas, also the powers of both cardiovascular impulses MREG_CARD_ and MREG_BOLD_ venous waves were higher in sleep, c.f. Figure 2. These brain areas have increased slow delta EEG power during sleep, suggesting increased I/CSF clearance based on animal studies (Ding et al., 2016; Helakari et al., 2022). Moreover, intrathecal gadolinium MRI contrast agent and protein solutes accumulated around the same parasagittal areas in human glymphatic studies (Ringstad and Eide, 2020; Albayram et al., 2022).

Based on the spatial overlaps, we believe that the increased synchrony between the arterial MREG_CHe_ and venous MREG_BOLD_ during sleep reflects altered fluid convection between the two compartments, a phenomenon detected using these two MREG contrasts (as explained in detail in the Introduction). This finding could reflect the increased power of the pulsations in these brain areas. The increased synchrony between the arterial and venous pulsations could suggest that a local mechanism allows pulsations to pass more readily from the arterial to the venous side, via increased capillary blood flow and recruitment. However, it may also *partially* indicate increased water flow from periarterial CSF space over the glia limitans into perivenous space. During sleep, vasomotion intensifies, causing significant fluctuations that impact both arterial and venous compartments (Bojarskaite et al., 2023). The sagittal sinus, being a large blood reservoir, may experience a more pronounced influence. Addressing this issue requires more detailed investigations utilizing microscopic methods and tools specifically tailored to detect water exchange and blood–brain barrier permeability.

### Venous BOLD fluctuations

Human sleep manifests in strong increases in the VLF < 0.1 Hz BOLD signal fluctuations (Fukunaga et al., 2006; Horovitz et al., 2008; Liu et al., 2018; Fultz et al., 2019). In the awake state, the functional BOLD signal exhibits robust coupling with neuronal activity (Ogawa et al., 1992; Biswal et al., 1995; Fox et al., 2005; Drew et al., 2008; Rayshubskiy et al., 2014). However, in sleep (Fukunaga et al., 2006; Horovitz et al., 2008; Helakari et al., 2022; Picchioni et al., 2022), or anesthesia (Kiviniemi et al., 2000, 2005; Ma et al., 2016), this local coupling seems to be dominated by slowly propagating 0.03–0.04 Hz waves (Kiviniemi et al., 2016; Yousefi et al., 2018; Gu et al., 2021; Raut et al., 2021). While some researchers argue that neurovascular coupling is maintained in sleep (Fultz et al., 2019), others showed that a 0.04 Hz vasomotor wave appears under anesthesia, which masks the signals that were functionally coupled to neuronal activity (Ma et al., 2016). Consistent with these findings, a recent study presented compelling evidence of the simultaneous influence of these two signaling mechanisms on BOLD signals in the human brain. One mechanism propagates into a vasomotor wave type, while the other establishes functional connectivity with a standing wave type coupled to neuronal activity (Bolt et al., 2022).

A recent study on mice indicates that innervations from the locus coeruleus produce slow noradrenaline-level waves that drive widespread vasomotor waves at around 0.03 Hz to control sleep state architecture (Kjaerby et al., 2022). Human data show that, in a state of reduced consciousness, the brain exhibits slow co-activation patterns induced by the cholinergic nucleus basalis of Meynert (Liu et al., 2018), whereas an invasive study in monkeys showed that detection of this coactivation in fMRI data is encumbered by physiological pulsations (Chang et al., 2016). Those studies align with ultrafast MREG measurements showing an increase in slow VLF changes in sleep, where the respiratory and cardiac pulsations similarly increased according to a separate mechanism during sleep (Helakari et al., 2022).

Waves involved in the regulation of blood pressure in the ~0.1 Hz range may explain much of the BOLD contrast signal in awake humans (Whittaker et al., 2019; Attarpour et al., 2021). The present study shows that during NREM sleep, the slower variations appear both in the global BOLD signal and the peripheral signal, especially in the <0.05 Hz frequency range (Figure 2B). The increase in power in the peripheral vasomotor power occurs in the same frequency ranges as were seen globally in the brain. In both regions, strongly dominant waves appear in the ~0.02 and 0.04 Hz ranges. It has been shown that spectral analysis of the mean flow velocity of the middle cerebral artery increases in power at 0.02 Hz during NREM sleep (Lee et al., 2019). Sleep also increases in power at 0.05 Hz (Fultz et al., 2019). In the VLF range, there are individual frequency peaks similar to the cardiac and respiration ranges. It should be noted that cardiorespiratory aliasing occurs significantly in the VLF range when TR is more than 100 ms (Huotari et al., 2019). Because of this, we suggest that the VLF power was measured more accurately in this paper. Indeed, we propose that sleeping hemodynamic waves appear to be associated with vasomotion and that <0.1 Hz vasomotor waves may explain much of the BOLD signal during NREM N1-N2 sleep; we infer that this may be a significant drive of fluid transport. Furthermore, as the vasomotor wave intensifies, there is an associated acceleration of tracer movement along the perivascular space (van Veluw et al., 2020).

Results of this study show that in NREM sleep, part of the VLF signal indeed originates in a systemic-origin vasomotor wave. Furthermore, the increase in the vasomotor wave power and synchrony appearing in the human brain in NREM sleep may significantly participate in the increased brain fluid transport in sleep. The default mode network seems to start following the peripheral vasomotor waves supporting earlier evidence on the modulatory role of it in changes of consciousness. In addition, the venous signal harmonizes with the arterial signal in parasagittal brain areas during sleep, which may also enable CSF flow. Finally, we find that cardiac beat-to-beat pulsatility is not coherent with peripheral pulsatility during NREM sleep. With the advent of fast fMRI techniques, it is now possible to visualize the drivers of CSF flow. This study provides additional information on how cardiovascular changes occurring in NREM sleep affect brain pulsations, which implies that aspects of cerebral circulation could play a significant role in driving brain fluid transport.

### Limitations

25 subjects participated in the study (Helakari et al., 2022), but complete data including sleep scores, MREG, and PPG data for both awake and sleep scans were available for only 11. Exclusion criteria primarily resulted from continuous artifacts in EEG, and a detailed account is provided in Helakari et al. (2022). Data from one subject were also excluded because of excessive noise in the PPG signal, resulting in a total number of 10 subjects.

Our objective was to investigate the similarity of signals at the same phase in awake and NREM sleep states. Because we wanted to study similarity and phase locking, we ruled out consideration of a negative correlation, which may be a matter for future investigation. Also, since the cardiac power increase during sleep was smaller compared to the VLF and respiratory pulsations changes, it could be proposed that the modulating factor affecting both CHe and BOLD signals could be the respiratory pulsations, which are known to modulate the cardiovascular signal, whereas the veins mediate the respiratory effects (Raitamaa et al., 2019, 2021; Helakari et al., 2022). In this regard, it is noteworthy that the peripheral arterial amplitude vasomotion (PPG_CHe_) and volume (PPG_VLF_) fluctuations and the venous MREG_BOLD_ signal become markedly more synchronous in sleep than with the intracranial arterial amplitude MREG_CHe_. The venous BOLD lag also increased significantly compared to peripheral blood volume PPG_VLF_ oscillations (Figure 5). One might expect that the intracranial arterial CHe modulations should be more in synchrony with the venous BOLD signal than with peripheral signals, but the intracranial synchrony seen in the present study appear to be confined to areas showing sleep-related increases in solute transport (Ringstad and Eide, 2020), c.f. Figure 4. Thus, the synchrony of local and peripheral influences must be considered together.

The awake scan was measured at 4:00–6:00 P.M. and the sleep scan at 6:00–8:00 A.M. Circadian rhythms may have confounded our results but to minimize that possibility, we selected for analysis those continuous 5-min segments that included the highest amount of NREM N2 sleep according to AASM sleep scoring criteria. We also used 24-h sleep deprivation to increase sleep pressure and help subjects fall asleep in the scanner (Portas et al., 2000; Horovitz et al., 2009). Deprivation can lead to microvascular dysfunctions (Sauvet et al., 2010) and may impact the findings.

## Data Availability

The datasets presented in this article are not readily available because privacy or ethical restrictions. Requests to access the datasets should be directed to JT, johanna.tuunanen@oulu.fi.

## Footnotes

^1^ ouraring.com
