# Supplementary material for: Cardiovascular and vasomotor pulsations in the brain and periphery during awake and NREM sleep in a multimodal fMRI study
Source: Front Neurosci. 2024 Oct 8;18:1457732. doi: 10.3389/fnins.2024.1457732 (PMC11493778; doi:10.3389/fnins.2024.1457732)
Supplement: Supplementary file 1 [file Data_Sheet_1.DOCX]

Supplemental file for Tuunanen et al. ”Cardiovascular and vasomotor pulsations in the brain and periphery during awake and NREM sleep in a multimodal fMRI study”.


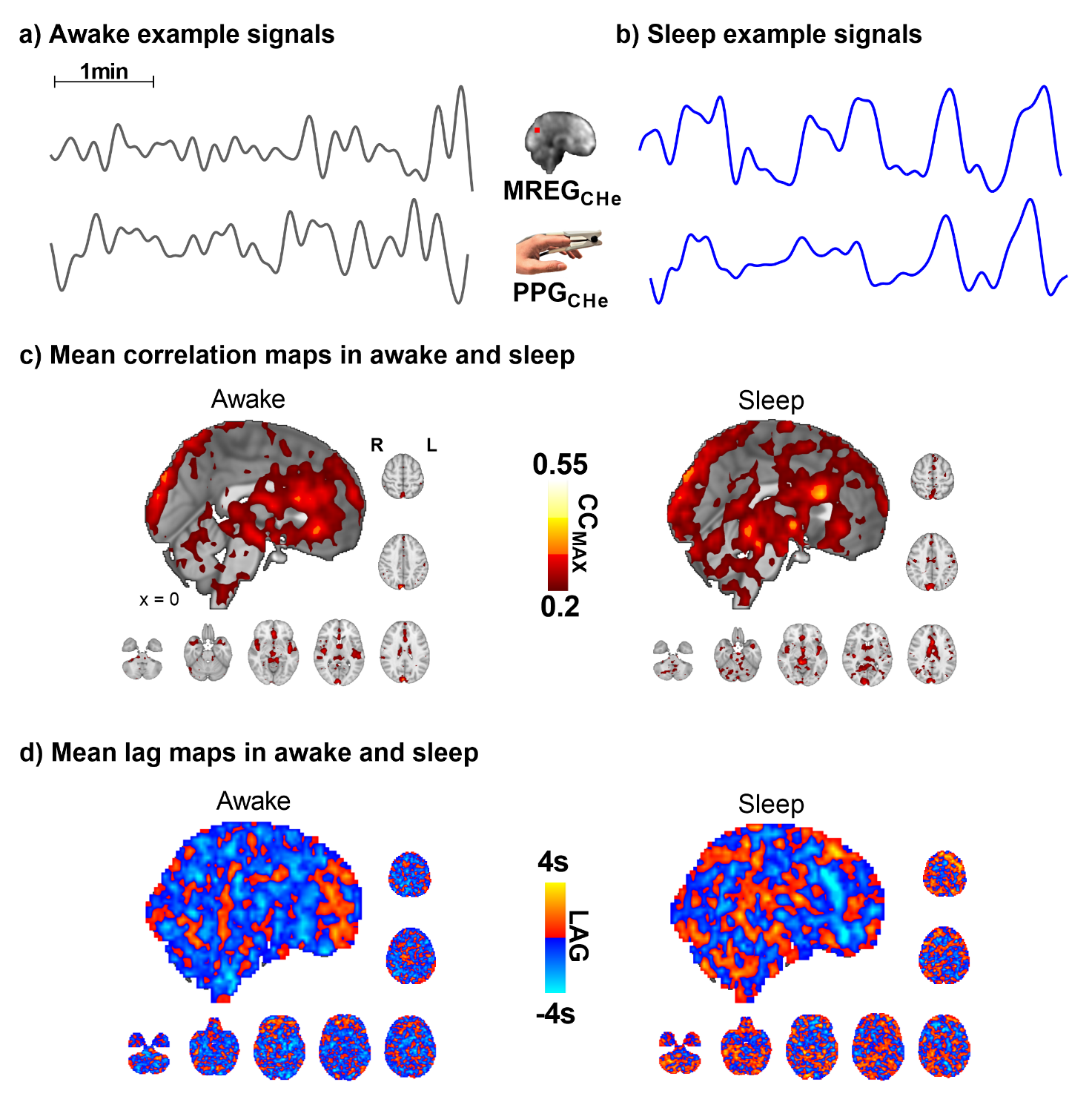

**Supplementary Figure 1.** Correlation between cerebral and peripheral cardiac hemodynamic envelope signals (n = 10). a-b) Representative signals of MREG_CHe_ and PPG_CHe_ during awake and sleep states. c) The synchrony was highest in arterial and sinus sagittal areas, without significant changes between awake and sleep states. d) There are no significant changes in lag values between awake and sleep recordings.
